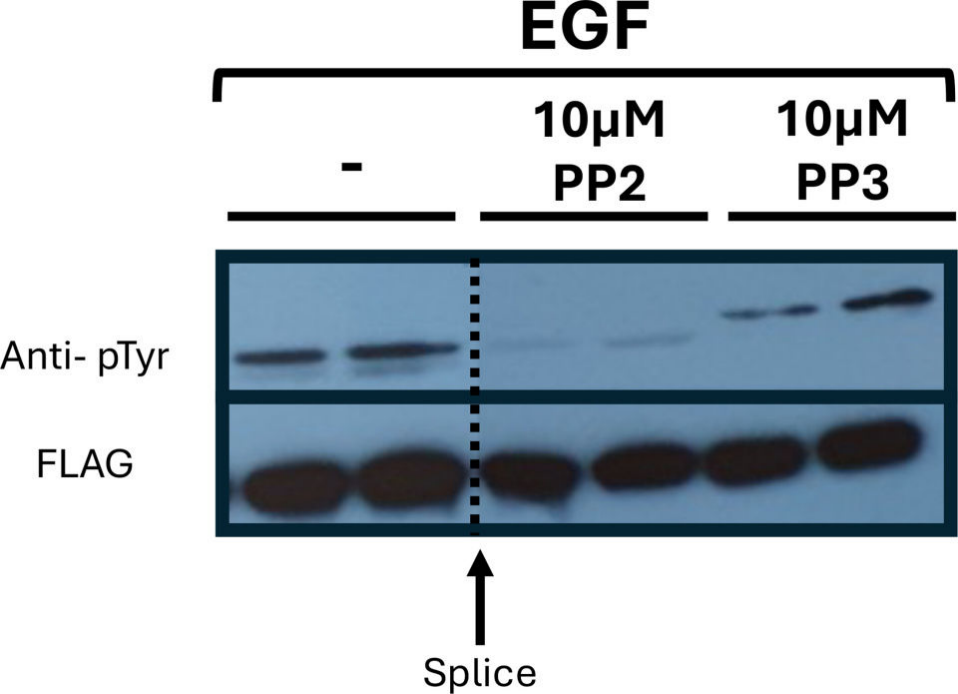# Correction: Phosphoinositide 3-kinase-dependent phosphorylation of the dual adaptor for phosphotyrosine and 3-phosphoinositides by the Src family of tyrosine kinase

**DOI:** 10.1042/BCJ3490605_COR

**Published:** 2025-10-09

It has come to the attention of the authors of the article “Phosphoinositide 3-kinase-dependent phosphorylation of the dual adaptor for phosphotyrosine and 3-phosphoinositides by the Src family of tyrosine kinase” (DOI: 10.1042/bj3490605) that there is a repetition of blots representing FLAG (EGF panel) in Figure 1C and FLAG (EGF panel) in Figure 3A. This was an unintentional error made during the assembly of the figure.The raw data for Figures 1C and 3A have been identified, and it is confirmed that the incorrect data were used for Figure 3A. A new Figure 3A (EGF panel), containing the correct data, is provided here.

The raw data and requested correction have been assessed by, and agreed with, the Publisher. The authors apologise for the error and any inconvenience this may have caused. The data analysis and conclusions are not affected by this error.

**Figure 3A EGF:**